# Distinct Biomarker Profiles of B-Cell Activation in Metabolic and Viral Hepatic Fibrosis

**DOI:** 10.3390/ijms26135942

**Published:** 2025-06-20

**Authors:** Umberto Basile, Valeria Carnazzo, Valerio Basile, Stefano Pignalosa, Francesca D’Ambrosio, Ilaria Vinante, Marzia Tagliaferro, Benedetta Niccolini, Riccardo Di Santo, Gian Ludovico Rapaccini, Enrico Rosa, Marco De Spirito, Mariapaola Marino, Gabriele Ciasca

**Affiliations:** 1 Department of Clinical Pathology, Santa Maria Goretti Hospital, 04100 Latina, Italy; u.basile@ausl.latina.it (U.B.); v.carnazzo@ausl.latina.it (V.C.); s.pignalosa@ausl.latina.it (S.P.); i.vinante@ausl.latina.it (I.V.); m.tagliaferro@ausl.latina.it (M.T.); 2 Clinical Pathology Unit and Cancer Biobank, Department of Research and Advanced Technologies, Regina Elena National Cancer Institute I.R.C.C.S., 00144 Rome, Italy; valeriobasile90@gmail.com; 3 Department of Laboratory and Infectious Diseases Sciences, Fondazione Policlinico Universitario “A. Gemelli” I.R.C.C.S., 00168 Rome, Italy; francesca.dambrosio@policlinicogemelli.it; 4 Dipartimento di Neuroscienze, Sezione di Fisica, Università Cattolica del Sacro Cuore, 00168 Rome, Italy; benedetta.niccolini@policlinicogemelli.it (B.N.); enrico.rosa.pz@gmail.com (E.R.); marco.despirito@unicatt.it (M.D.S.); gabriele.ciasca@unicatt.it (G.C.); 5 Fondazione Policlinico Universitario “A. Gemelli” I.R.C.C.S., 00168 Rome, Italy; gianludovico.rapaccini@policlinicogemelli.it; 6 Department of Life Science, Health and Health Professions, Link Campus University, 00165 Rome, Italy; r.disanto@unilink.it; 7 Dipartimento di Medicina e Chirurgia Traslazionale, Sezione di Patologia Generale, Università Cattolica del Sacro Cuore, 00168 Rome, Italy

**Keywords:** free light chains, BAFF, IgG subclasses, MAFLD, HCV, biomarkers, fibrosis

## Abstract

Increasing evidence underlines the role of B-cells in the development of hepatic fibrogenesis following viral infections and metabolic dysfunction, through different mechanisms depending on the etiology. Circulating biomarkers of B-cell activation—such as B-cell activating factor (BAFF), immunoglobulin G (IgG) subclasses, and free light chains (FLCs)—may be associated with different results between viral and metabolic hepatic fibrosis, supporting their use as diagnostic tools. We conducted a case-control study including 100 patients with liver fibrosis, 50/100 of metabolic etiology and 50/100 of viral etiology. A reference group of 30 healthy donors was included as control. Serum levels of BAFF were measured using ELISA, while IgG subclasses (IgG1, IgG2, IgG3, IgG4), κ-FLC, λ-FLC, and the κ/λ ratio were quantified by turbidimetric methods. In univariate analysis, κ-FLC, λ-FLC, and BAFF levels were significantly elevated in both patient groups, with the highest concentrations consistently observed in metabolic fibrosis. IgG2 was selectively increased in metabolic fibrosis, whereas IgG3 was specifically elevated in viral fibrosis. Multivariate analysis confirmed these findings, showing a clear clustering of the three groups and identifying increased BAFF and κ-FLC as key features of metabolic fibrosis, while elevated IgG3 emerged as the most distinctive marker of viral etiology. These results reveal distinct B-cell-related immunological signatures in metabolic and viral hepatic fibrosis supporting the role of BAFF, FLCs, and IgG subclasses as biomarkers of etiological differentiation, and provide novel insights into the immune mechanisms driving fibrosis progression, potentially contributing to the identification of new therapeutic targets.

## 1. Introduction

Hepatic fibrosis is a key determinant of disease progression in chronic liver diseases (CLD) [1,2,3,4,5]. In most cases, this condition results from sustained inflammatory stimuli that trigger the recruitment and activation of immune cells in the liver and promote the release of fibrogenic mediators, ultimately leading to tissue remodeling and loss of hepatic function. This inflammatory state can result from different etiologies, with metabolic dysfunction and chronic hepatitis C virus (HCV) infection being among the most prevalent [6,7,8,9]. In this context, Metabolic-Associated Fatty Liver Disease (MAFLD)—recently redefined from the former term Non-Alcoholic Fatty Liver Disease (NAFLD)—is the most common CLD worldwide, with an estimated global prevalence of 25% in adults. It encompasses a wide spectrum of conditions, ranging from simple steatosis to non-alcoholic steatohepatitis, cirrhosis, and hepatocellular carcinoma [7,10,11,12,13]. In parallel, chronic HCV infection remains a major contributor to liver fibrosis globally, despite the success of antiviral treatments. HCV is a lymphotropic virus that can stimulate B-cells and lead to systemic immune alterations, including extrahepatic manifestations such as mixed cryoglobulinemia [8,14,15,16,17,18].

Main diagnosis strategies to evaluate liver fibrosis take into account liver biopsy, imaging techniques, and a list of serum molecules easily tested in the laboratory. Along with classic clinical protocols measuring aspartate aminotransferase (AST), alanine aminotransferase (ALT), platelet count, albumin, and international normalized ratio (INR), extracellular matrix (ECM) components have been addressed as novel liver biomarkers, such as hyaluronic acid, laminin, collagen type III N-peptide (PIIIP N-P), and type IV collagen (C-IV), as well as other non-ECM emerging biomarkers, such as cholylglycine and Golgi protein-73 [19,20,21].

Given the central role of B-cell activation in both metabolic and viral liver fibrosis, we recognized it would be valuable to investigate circulating biomarkers associated with this process, including B-cell activating factor (BAFF), immunoglobulin G (IgG) subclasses, and free light chains (FLCs). Although the underlying mechanisms differ, B lymphocyte activation is believed to contribute to liver injury and fibrosis in both MAFLD and HCV-related disease [17,22,23]. In HCV, this activation is direct and virus-mediated [8,15,16,17,18,19], whereas in MAFLD it appears to be driven by metabolic and inflammatory cues rather than by a specific antigenic trigger. In this context, B-cells may contribute to hepatic inflammation by secreting pro-inflammatory mediators and promoting the activation of hepatic stellate cells (HSCs), which are key drivers of fibrogenesis [23,24,25,26].

BAFF is a cytokine that regulates peripheral B-cell maturation, survival, immunoglobulin production, and class-switch recombination [27]. Its levels are increased in chronic HCV infection and autoimmune conditions, reflecting persistent antigen-driven stimulation [22]. Increased BAFF has also been observed in MAFLD, where it may be induced by metabolic and inflammatory signaling contributing to liver injury and fibrotic progression [17,28].

IgG, the most abundant immunoglobulin isotype in serum, includes four subclasses (IgG1–IgG4) with distinct functional roles [29]. In HCV-related disease, oligo-clonal IgG3 with rheumatoid factor activity is increased, reflecting clonal expansion in response to persistent viral stimulation [30]. In MAFLD, altered IgG subclass profiles have also been reported, with prominent changes in IgG2; this subclass has been linked to insulin sensitivity and systemic inflammation [31,32,33,34].

Free kappa (κ-FLC) and lambda (λ-FLC) chains of Ig are produced by activated B-cells and are considered sensitive markers of B-cell activation. Due to their short half-life, they are considered more dynamic indicators of ongoing immune activation than total immunoglobulin levels [35]. Abnormal FLC levels have been linked to disease activity in several chronic inflammatory and autoimmune conditions [36,37,38,39], and recent studies suggest a role in the pathogenesis and severity of MAFLD [40,41].

Based on these premises, our aim was to investigate whether a panel of circulating B-cell-related markers—namely IgG subclasses, κ- and λ-FLCs, and BAFF—could identify distinct immune profiles across hepatic fibrosis with different etiologies. Specifically, in this study we recruited patients with MAFLD or HCV infection, along with healthy controls. Defining such immune fingerprints may contribute to better stratification of patients with liver fibrosis and provide insights into potential targets for immunomodulatory intervention.

## 2. Results

### 2.1. Differential Expression of B-Cell Activation Markers in Metabolic and Viral Fibrosis: Univariate and Adjusted Findings

In this study, we aimed to identify an inflammatory fingerprint of hepatic fibrosis from different etiologies, specifically metabolic and viral. To this end, we recruited 50 patients with hepatic fibrosis of metabolic origin (group M), 50 with viral fibrosis from HCV infection (group V), and 30 healthy controls (group C).

Table 1 reports the demographic (age and sex) and clinical characteristics, including serum FLCs, IgG subclasses, and BAFF, for each group. The median age differed significantly across groups (*p* = 0.002), with group V having the youngest participants (median: 52 years, IQR: 46.3–58.8). No significant differences in sex distribution were observed (*p* = 0.2). Similarly, the distribution of METAVIR scores did not differ significantly between the two pathological groups (*p* = 0.3).

**Figure 1:**
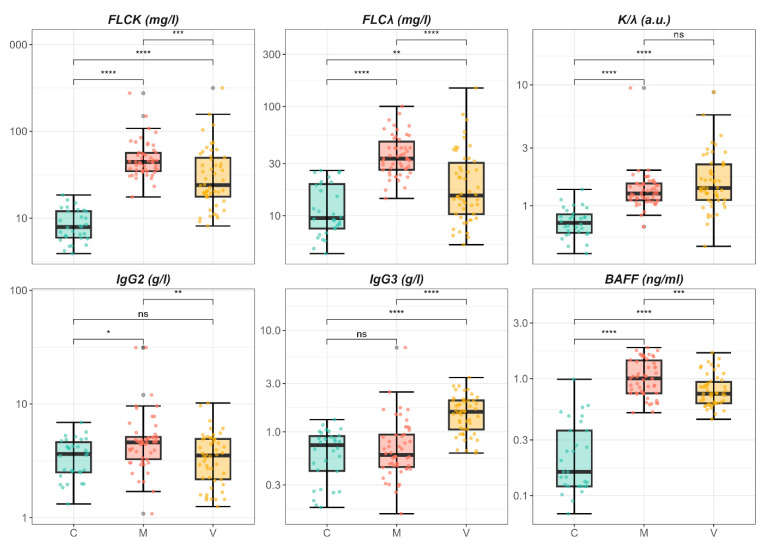
Boxplot analysis of significant parameters from Table 1, showing pairwise comparisons of kappa free light chains (k-FLC), lambda free light chains (λ-FLC), kappa-to-lambda ratio (k/λ), IgG2, IgG3, and BAFF among patients with metabolic fibrosis (group M), viral fibrosis (group V), and healthy controls (group C). Significance levels are denoted as follows: *p*  <  0.05 (*), *p*  <  0.01 (**), *p*  <  0.001 (***), *p*  <  0.0001 (****), not significant (ns).

Laboratory parameters showed several notable differences among groups, with some biomarkers showing highly significant variations (*p* < 0.001). To further explore these differences, we analyzed the biomarkers using boxplots (Figure 1), with post hoc pairwise comparisons performed using the Wilcoxon test corrected by Bonferroni. Only variables with an overall *p*-value < 0.01 in the Kruskal–Wallis test were included.

Specifically, κ-FLC and λ-FLC levels were significantly higher in patients with metabolic fibrosis compared to both viral fibrosis and healthy controls, with group V also showing elevated levels relative to controls. The κ/λ ratio was increased in both fibrosis groups compared to controls, although no significant difference emerged between group M and group V. Notably, only group V included a substantial proportion of individuals exceeding the normal reference range for this parameter (0.26–1.65). Regarding immunoglobulin subclasses, IgG2 levels were significantly higher in group M, whereas IgG3 levels were significantly elevated in group V compared to the other groups. BAFF levels also differed markedly among groups, being significantly increased in both pathological groups compared to controls, with the highest median value observed in group M.

**Figure 2:**
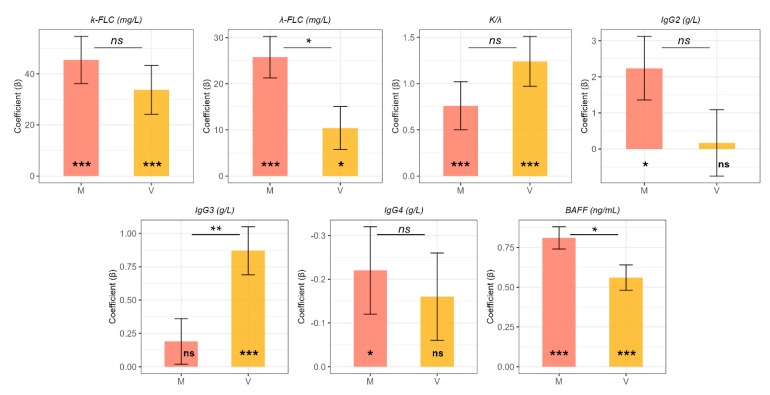
Age- and gender-adjusted differences (coefficients β) for biochemical markers comparing metabolic fibrosis (M) and viral fibrosis (V) groups to controls. The coefficients represent adjusted differences from the control group, with error bars showing standard errors. Significant differences from the control group (C) are indicated by confidence intervals not crossing zero and by asterisks below each bar. Significance of the comparison between M and V groups is indicated above the bars. Significance levels are denoted as follows: *p*  <  0.05 (*), *p*  <  0.01 (**), *p*  <  0.001 (***), not significant (ns).

For the sake of completeness, given the differences in age observed across groups, in Table 2 we display the age- and gender-adjusted differences in biomarker levels between groups, using the control group as reference. These results are further illustrated in Figure 2, which shows adjusted estimates and standard errors derived from multivariable linear regression models.

Adjusted values confirmed most of the findings from the unadjusted analyses. κ-FLC levels were significantly increased in both group V (adjusted difference = 33.73 mg/L, *p* = 0.00057) and group M (adjusted difference = 45.42 mg/L, *p* < 0.0001) compared to controls. Similarly, λ-FLC levels were higher in group V (adjusted difference = 10.43 mg/L, *p* = 0.027) and group M (adjusted difference = 25.75 mg/L, *p* < 0.0001). The κ/λ ratio remained significantly elevated in metabolic fibrosis (group M: adjusted difference = 0.76, *p* = 0.004). IgG2 was confirmed to be significantly increased in group M (adjusted difference = 2.24 g/L, *p* = 0.012), while IgG3 was significantly higher in group V (adjusted difference = 0.87 g/L, *p* < 0.001), with no difference observed in group M. Adjusted values also revealed a statistically significant reduction in IgG4 levels in the metabolic fibrosis group (adjusted difference = −0.22 g/L, *p* = 0.023), which was not observed in the unadjusted comparisons.

### 2.2. Correlation Patterns and Insights into the Role of BAFF

**Figure 3:**
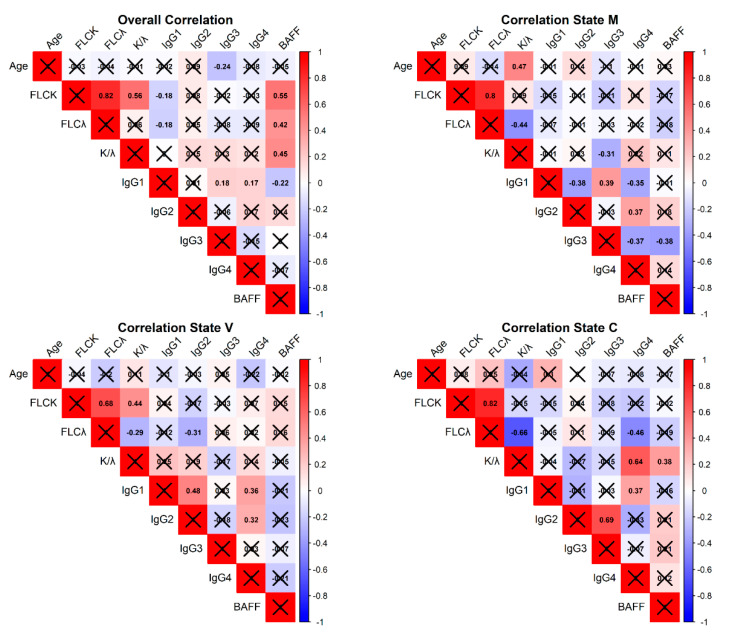
Pairwise Spearman correlation matrices among age, serum free light chains (κ-FLC, λ-FLC), κ/λ ratio, IgG subclasses, and BAFF levels. The analyses were conducted in the overall cohort (**top left**) and separately within each group: metabolic fibrosis (M, **top right**), viral fibrosis (V, **bottom left**), and healthy controls (C, **bottom right**). The color scale indicates the strength and direction of the correlation coefficient, ranging from −1 (blue, strong negative correlation) to +1 (red, strong positive correlation). Only statistically significant correlations (*p*  <  0.05) are shown with numerical values; non-significant associations are marked with a black “×”.

To better characterize the immunological relationships among the studied biomarkers, we analyzed pairwise Spearman correlations between age, free light chains (κ-FLC, λ-FLC), IgG subclasses, and BAFF, both overall and stratified by group (Figure 3). In the overall cohort, κ-FLC and λ-FLC levels were strongly correlated (r = 0.82), as expected. BAFF levels showed moderate positive correlations with κ-FLC (r = 0.55) and λ-FLC (r = 0.42). However, when the analysis was stratified by group, these correlations were no longer statistically significant. In the metabolic group, BAFF was positively correlated with IgG2 (r = 0.37) and IgG3 (r = 0.38), while in the viral and control groups, no significant correlations with BAFF were observed.

Attempting to clarify why the correlation between BAFF and FLCs was lost in the stratified analyses, we performed two multivariable linear regression models using κ-FLC or λ-FLC as the outcome. Each model included fibrosis etiology (State: control, metabolic, or viral) and BAFF levels as independent variables. As reported in Table 3, fibrosis etiology was significantly associated with FLC levels. κ-FLC levels were higher in both the viral and metabolic groups compared to controls (State V: β = +35.80, *p* = 0.0017; State M: β = +49.61, *p* < 0.0001). Similarly, λ-FLC levels were elevated in the pathological groups (State V: β = +12.76, *p* = 0.0218; State M: β = +26.87, *p* < 0.0001). In contrast, BAFF was not a significant independent predictor of κ-FLC or λ-FLC levels. We also tested the inclusion of interaction terms between BAFF and fibrosis etiology, but these were not statistically significant and did not improve model fit.

### 2.3. Multivariate Analysis of Circulating Markers for Hepatic Fibrosis: A Molecular Fingerprint of Viral and Metabolic Etiology

**Figure 4:**
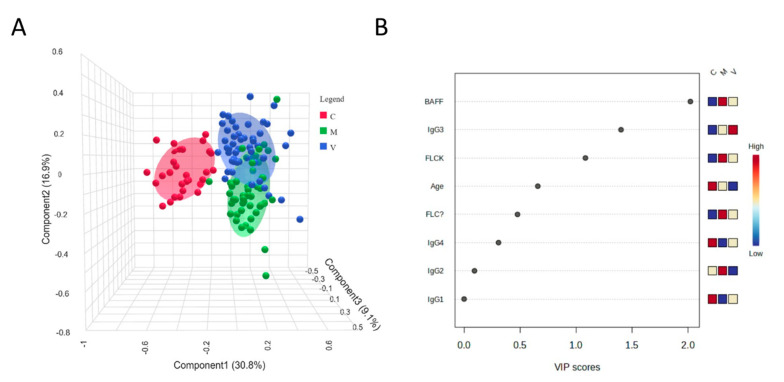
(**A**) Three-dimensional score plot from partial least squares discriminant analysis (PLS-DA), illustrating the distribution of subjects based on immune marker profiles across the three groups: healthy controls (C, red), metabolic fibrosis (M, blue), and viral fibrosis (V, green). (**B**) variable importance in projection (VIP) scores summarizing the contribution of each variable to the PLS-DA model. Heatmap bars on the right display the relative expression levels of each marker across the three groups (C, M, V), from low (blue) to high (red).

To gain further insight into the combined behavior of circulating biomarkers, we applied a supervised multivariate classification model using partial least squares discriminant analysis (PLS-DA). The three-dimensional score plot (Figure 4A) revealed a clear separation between the control group (C, red), the metabolic fibrosis group (M, blue), and the viral fibrosis group (V, green), with limited overlap among clusters. The robustness of this clustering was confirmed by permutation testing (*p* < 0.001), supporting the existence of distinct molecular signatures associated with each condition.

The variable importance in projection (VIP) scores, shown in Figure 4B, identified BAFF, κ-FLC, and IgG3 as the most relevant contributors to group separation (VIP ≥ 1). Both BAFF and κ-FLC levels were markedly elevated in the metabolic group, indicating that they represent key components of its inflammatory signature. In contrast, IgG3 levels were highest in viral fibrosis, supporting its role as a distinguishing feature of this etiology.

## 3. Discussion

Liver fibrosis is a common point of no return of chronic liver injuries of different etiologies, including viral infections and metabolic dysfunctions. Its progression involves complex immune and inflammatory mechanisms that may differ across underlying causes. MAFLD arises in the context of obesity, insulin resistance, type 2 diabetes, and dyslipidemia with contributing factors including low-grade inflammation, oxidative stress, and altered cytokine signaling [42,43,44,45]. In HCV-related disease, viral persistence promotes fibrosis by disrupting the balance between immunostimulatory and inhibitory cytokines, leading to hepatocellular damage and extracellular matrix deposition.

Chronic immune stimulation plays a key role in fibrogenesis, involving both innate and adaptive responses [19,46,47]. Among innate pathways, the activation of inflammasome contributes to inflammation by inducing IL-1β and IL-18 release, which promotes hepatic stellate cell activation and fibrotic remodeling [48,49]. In MAFLD, B-cell activation has been linked to cytokine-mediated regulation of intrahepatic immune cells and increased hepatic BAFF expression [23]. In HCV infection, the virus’s lymphotropic properties lead to frequent mono- or polyclonal expansion of B lymphocytes, consistent with chronic antigenic stimulation [17,18].

In this study, we investigated B-cell-related immune markers—BAFF, FLCs, and IgG subclasses—in patients with moderate fibrosis (Metavir F2–F3) of either metabolic or viral origin. These markers differed significantly from healthy controls and showed distinct patterns across etiologies (Figure 1). κ-FLC and λ-FLC levels were elevated in both pathological groups, with significantly higher values in metabolic fibrosis. These findings are consistent with previous reports of FLC elevations in chronic inflammatory and autoimmune diseases, where they reflect increased B-cell activity and immune dysregulation [35,36,37,38,39]. Although the κ/λ ratio was similarly increased in both groups, out-of-range values were more frequent in viral fibrosis, potentially indicating clonal B-cell expansion in the setting of chronic HCV infection [17,18].

The analysis of IgG subclasses offered additional information. IgG3 was elevated in viral fibrosis, in line with prior evidence that IgG3 can self-aggregate and exhibit rheumatoid factor activity in chronic HCV infection. These features may help identify cases where viral stimulation drives immune activation and, in some patients, an autoimmune drift [15,30]. Conversely, IgG2 was selectively increased in metabolic fibrosis, consistent with previous observations linking this subclass to immune responses against polysaccharide antigens and low-grade inflammation in metabolic disease [33,34]. BAFF was elevated in both groups, with higher values in metabolic fibrosis. This agrees with studies showing that BAFF is upregulated in both chronic HCV and autoimmune liver diseases, as well as in MAFLD, where inflammatory and metabolic stimuli may drive its expression and promote immune cell infiltration in the liver [50,51]. The clinical relevance of BAFF has also been supported by prior studies in HBV-related hepatocellular carcinoma, where elevated plasma BAFF levels were independently associated with tumor progression, invasiveness, and overall survival [52,53]. These findings highlight the broader immunopathological role of BAFF in liver disease and support its involvement not only in inflammation but also in fibrosis progression. In our study, the selective increase of BAFF in metabolic fibrosis suggests that it may represent a potential target for tailored therapeutic strategies in MAFLD-related fibrosis [54,55,56]. More interestingly and from a pathogenetic point of view, our result fits well with the suggested causative role of BAFF in enhancing adipogenesis and visceral adipose tissue inflammation as a new adipokine, interfering with the pathway of insulin-induced signaling, and accelerating systemic insulin resistance [57,58]. The liver plays a central role in regulating glycemia and, at the same time, in inducing dysglycemia: fatty changes occurring in liver will result in the development to insulin resistance; similarly, preliminary evidence suggests that improvement of NAFLD is associated with a decreased incidence of Type 2 Diabetes [59,60].

BAFF is a known marker of B-cell activation, and B-cells are also major producers of FLCs [22,27,35,36]. In the overall cohort, BAFF and FLCs were positively correlated (Figure 3), supporting a functional link between the two. However, this correlation was not maintained when analyzing each group separately. These discrepancies suggest that the apparent associations observed in the overall cohort may be driven by between-group shifts in biomarker levels, rather than reflecting within-group mechanistic relationships.

An alternative explanation, however, is purely statistical: the loss of significance in stratified analyses may simply reflect the reduced sample size within each group, which directly limits the power to detect significant correlations. To better disentangle these possibilities, we performed a multivariable regression analysis adjusting for fibrosis etiology (Table 3). In this model, BAFF was not independently associated with either κ or λ FLC levels, suggesting that the observed correlation in the overall cohort may be driven by differences between groups, rather than a direct biological effect.

However, our data do not allow us to rule out a direct association between BAFF and FLCs. This lack of association might simply reflect the reduced variability of both BAFF and FLC levels within each disease group, as compared to the wider variation observed across groups. In this context, more subtle fluctuations in BAFF—potentially linked to equally subtle changes in FLC production—may be obscured by biological noise and measurement variability. Clarifying this relationship will require larger, etiology-specific cohorts specifically designed to detect these finer-scale associations.

To explore whether a more integrated immune signature could distinguish the two etiologies, we applied a multivariate classification model using partial least squares discriminant analysis (PLS-DA) [61,62,63]. This method is particularly suitable for identifying patterns in datasets where variables are interrelated, such as in immune marker panels. Unlike univariate comparisons, PLS-DA identifies the combination of variables that best separates predefined groups, helping to define a disease-specific fingerprint. In our model, the clustering by fibrosis etiology was well defined, with BAFF, κ-FLC, and IgG3 emerging as the strongest contributors to group separation.

We further characterized this fingerprint by calculating variable importance in projection (VIP) scores, which measure the relative contribution of each variable to the model. BAFF and κ-FLC were the most relevant markers in the metabolic group, while IgG3 was the key variable associated with viral fibrosis. Together, these three markers form a compact immune signature reflecting both common and etiology-specific features of B-cell activation.

It is also important to acknowledge several limitations of the present study. First, its case-control design does not allow the assessment of biomarker dynamics over time or their potential correlation with disease progression or treatment response. Second, liver fibrosis was evaluated using transient elastography rather than histological staging, which may limit sensitivity in detecting early-stage disease. Third, while the sample size was sufficient to detect major group-level differences, it may not have provided adequate power to explore more subtle intra-group associations or interactions. Furthermore, our analysis focused exclusively on circulating biomarkers, without including phenotypic or functional characterization of B-cell subpopulations. Considering these limitations, future studies should aim to include larger, multicentric cohorts and adopt a longitudinal design to validate and expand upon the present findings, while also integrating cellular and functional immune profiling, to clarify the role of identified biomarkers in the pathogenesis of metabolic and viral fibrosis.

In conclusion, we identified a B-cell-related immunological fingerprint composed primarily of BAFF, κ-FLC, and IgG3. Specifically, elevated BAFF and κ-FLC levels characterized metabolic fibrosis, while increased IgG3 was distinctive of viral fibrosis. This pattern suggests that selected circulating immune markers can reflect underlying disease mechanisms and provide meaningful stratification of patients by etiology. Such immune profiling may also offer a valuable starting point for the development of tailored therapeutic strategies in chronic liver disease.

## 4. Material and Methods

### 4.1. Patients and Methods

In this retrospective study were consecutively enrolled 100 patients, divided into 2 groups, as follows: 50 patients with MAFLD, and 50 HCV positive patients, from January 2018 to December 2021 referring at the Department of Gastroenterology, Fondazione Policlinico Universitario “A. Gemelli”, I.R.C.C.S., in Rome (Italy). Moreover, 30 healthy blood donors (HBD) were used as negative controls. Laboratory routine assays for alanine aminotransferase (ALT), aspartate aminotransferase (AST), γ-glutamiltransferase (GGT), glucose, insulin, lipid profile, and alkaline phosphatase (AP) were performed. MAFLD patient group included subjects with age ≥ 18, both genders; instrumental measurement of FibroScan (Echosens, Paris, France) was carried out to obtain liver stiffness measurement (LSM) and Controlled Attenuation Parameter (CAP). Before the examination, patients were required to fast for at least 8 h. Measurements of liver stiffness with transient elastography in these patients were well correlated with fibrosis METAVIR stages.

HCV-positive patient group included subjects age ≥ 18, both genders; instrumental measurement of FibroScan was like first cohort.

Exclusion criteria: presence of decompensated cirrhosis and/or Child Pugh B or C, average alcohol consumption ≥ 20/30 g/day (males/females), active chronic viral hepatitis (HBV, HCV, or HIV), other known chronic liver diseases (autoimmune hepatitis, biliary diseases, hemochromatosis, Wilson disease, alfa-1 deficiency disease). For this study, we also excluded patients with autoimmune diseases, inflammatory bowel diseases, blood-related oncological disease of any type, cancer (primary or secondary) within 5 years before enrollment.

A subset of 30 control samples was obtained from HBD who had previously been tested for the absence of monoclonal components by serum protein electrophoresis and serum/urine immunofixation electrophoresis, with a negative C-reactive protein (CRP) result, and displaying a transient elastography having F0 METAVIR score.

### 4.2. Laboratory Testing

The collected samples were centrifuged at 2500 g for 10 min and serum divided in aliquots before being frozen at −80 °C and stored until analysis. Samples were thawed only once, keeping them at room temperature and immediately analyzed. The analysis was performed by an operator without knowledge of the clinical history of the samples.

Serum FLCs were assessed using the Freelite™ Human Kappa and Lambda Free Kits (The Binding Site, Birmingham, UK) on an Optilite instrument (The Binding Site, Birmingham, UK; free κ normal range: 3.3–19.4 mg/L; free λ normal range: 5.7–26.3 mg/L). A ratio of k/λ < 0.26 or >1.65 was considered abnormal according to the manufacturer’s recommendations.

IgG subclasses levels were measured by turbidimetry through the employment of Human IgG and IgG subclass liquid reagent kits (The Binding Site Birmingham, UK) with Optilite instrument according to the manufacturer’s recommendations. Concentrations are automatically calculated by reference to a standard curve stored within the instrument. Reference range for subclasses: 3.82–9.29 g/L for IgG1; 2.42–7.0 g/L for IgG2; 0.22–1.76 g/L for IgG3; 0.04–0.86 g/L for IgG4. Samples were tested according to the manufacturer’s instructions, and serum dilutions, where necessary, were performed according to the manufacturer’s recommendations.

Circulating levels of BAFF were measured using a Human BAFF Quantikine ELISA Kit (R&D Systems, Inc, Minneapolis, MN, US); the kit employs an anti-BAFF antibody pre-coated onto a 96-well microtiter plate (capture antibody). Following incubation with standards/test samples, wells were washed and then incubated with biotinylated anti-BAFF antibody (detection antibody), which binds the captured BAFF present in each well. Following incubation, unbound biotinylated antibody was removed by washing, and an HRP-Streptavidin conjugate was added to the wells. Following incubation and washing, the addition of TMB substrate solution allows visualizing the HRP enzymatic reaction by catalysis producing a blue-colored product that changes to yellow after addition of acidic stop solution. The concentration of BAFF has been calculated by reading the O.D. absorbance at 450 nm in a microplate reader and referring to the standard curve.

### 4.3. Statistical Methods

All statistical analyses and data visualizations were performed using R (version 4.4.1). Quantitative variables were tested for normality using the Shapiro–Wilk test and by observing Q-Q plots. Significant deviations from normality were found; hence, quantitative variables are reported as medians with interquartile ranges (IQR). Tabular data were presented using the gtsummary package [64]. Group comparisons were performed using the Kruskal–Wallis test, followed by post hoc pairwise comparisons with Bonferroni correction. Qualitative data are reported in terms of counts and percentages, and groups were compared using the chi-square test or Fisher’s exact test, as appropriate. Age- and gender-adjusted differences were computed using multivariable linear regression, with age and gender as covariates and laboratory parameters as dependent variables. Controls were used as the reference group. Correlations between laboratory markers and demographic variables were assessed both in the overall dataset and within each group using Spearman’s rank correlation coefficient. To this end, correlation coefficients were organized into correlation matrices, as described elsewhere. Multivariate analysis of the circulating markers in relation to group classification was performed using partial least squares discriminant analysis (PLS-DA) with variable importance in projection (VIP) scores, implemented through the MetaboAnalyst platform [61,65,66].

## Figures and Tables

**Table 1:** Demographic and clinical characteristics of patients. Age, gender, IgG subclasses, serum free light chains (FLCs), and B-cell activating factor (BAFF) of patients with metabolic fibrosis (group M), viral fibrosis (group V), and healthy controls (group C) are reported.

Characteristic	C, N = 30 ^1^	M, N = 50 ^1^	V, N = 50 ^1^	*p*-Value ^2^
Age	60.0 (54.3, 64.0)	58.0 (53.3, 66.8)	52.0 (46.3, 58.8)	0.002
Sex	16.0 (53.3%)	28.0 (56.0%)	19.0 (38.0%)	0.2
FLC-k	7.9 (6.0, 12.0)	44.3 (34.8, 56.5)	24.1 (17.7, 49.7)	<0.001
FLC-λ	9.5 (7.6, 19.5)	33.4 (26.4, 47.8)	15.3 (10.3, 30.5)	<0.001
K/λ	0.7 (0.6, 0.8)	1.3 (1.1, 1.5)	1.4 (1.1, 2.2)	<0.001
IgG1	8.0 (5.9, 8.5)	5.7 (4.3, 7.1)	6.8 (4.8, 9.9)	0.061
IgG2	3.6 (2.5, 4.6)	4.6 (3.3, 5.1)	3.5 (2.2, 4.9)	0.01
IgG3	0.7 (0.4, 0.9)	0.6 (0.5, 0.9)	1.6 (1.1, 2.0)	<0.001
IgG4	0.3 (0.2, 0.8)	0.3 (0.2, 0.5)	0.3 (0.2, 0.7)	0.5
METAVIR-SCORE				0.3
F2		22.0 (44.0%)	28.0 (56.0%)	
F3		28.0 (56.0%)	22.0 (44.0%)	
BAFF	0.2 (0.1, 0.4)	1.0 (0.8, 1.4)	0.7 (0.6, 0.9)	<0.001

^1^ Median (IQR); n (%), ^2^ Kruskal–Wallis rank sum test; Pearson’s chi-squared test; Fisher’s exact test.

**Table 2:** Adjusted differences in biochemical markers for metabolic fibrosis (group M) and viral fibrosis (group V) compared to controls (group C). Age and gender were included as covariates in a multivariable linear regression model. The table presents the adjusted coefficients for fibrosis status (metabolic or viral), along with standard errors and *p*-values, using the control group as the reference.

	Group V		
Model	Adjusted Differences	Std. Error	*p*-Value
k-FLC (mg/L)	33.73	9.54	<0.001
λ-FLC (mg/L)	10.43	4.66	0.027
k/λ	1.24	0.27	<0.001
IgG2 (g/L)	0.17	0.92	0.85
IgG3 (g/L)	0.87	0.18	<0.001
IgG4 (g/L)	−0.16	0.10	0.11
BAFF (ng/mL)	0.56	0.08	<0.001
	**Group M**		
Model	Adjusted Differences	Std. Error	*p*-Value
k-FLC (mg/L)	45.42	9.21	<0.001
λ-FLC (mg/L)	25.75	4.5	<0.001
k/λ	0.76	0.26	<0.001
IgG2 (g/L)	2.24	0.88	0.012
IgG3 (g/L)	0.19	0.17	0.28
IgG4 (g/L)	−0.22	0.10	0.023
BAFF (ng/mL)	0.81	0.07	<0.001

**Table 3:** Multivariable linear regression models evaluating the association of BAFF and fibrosis etiology (State) with κ-FLC and λ-FLC levels (without interaction terms). The table reports regression coefficients (Estimate), standard errors (SE), and *p*-values. The reference group is the control group (C). The overall model *p*-value and adjusted R^2^ are also provided.

Model Outcome	BAFF (Estimate ± SE, *p*)	State V (Estimate ± SE, *p*)	State M (Estimate ± SE, *p*)	Model *p*-Value	Adj. R^2^
κ-FLC	–5.41 ± 11.25 *p* = 0.63	35.80 ± 11.14 *p* = 0.0017	49.61 ± 12.89 *p* < 0.0001	4.15 × 10⁻⁵	0.146
λ-FLC	–1.12 ± 5.55 *p* = 0.84	12.76 ± 5.49, *p* = 0.0218	26.87 ± 6.36 *p* < 0.0001	1.03 × 10⁻⁶	0.196

## Data Availability

The data presented in this study are available upon reasonable request to the corresponding author.

## Abbreviations

Chronic liver diseases (CLD), hepatitis C virus (HCV), Metabolic-Associated Fatty Liver Disease (MAFLD), Non-Alcoholic Fatty Liver Disease (NAFLD), hepatic stellate cells (HSCs), B-cell activating factor (BAFF), immunoglobulin G (IgG) subclasses, free light chains (FLCs), alanine aminotransferase (ALT), aspartate aminotransferase (AST), γ-glutamiltransferase (GGT), alkaline phosphatase (AP), liver stiffness measurement (LSM), controlled attenuation parameter (CAP), C-reactive protein (CRP).
